# Incidental Bystander or Essential Culprit: A Systematic Review of Bacterial Significance in the Pathogenesis of Breast Implant-Associated Anaplastic Large Cell Lymphoma

**DOI:** 10.3390/ijms25010355

**Published:** 2023-12-26

**Authors:** Jose A. Foppiani, Otakar Raska, Iulianna Taritsa, Angelica Hernandez Alvarez, Daniela Lee, Maria J. Escobar-Domingo, Josephine Berger, Pavel Klener, Kirsten A. Schuster, Daoud Abdo, Mark W. Clemens, Samuel J. Lin

**Affiliations:** 1 Division of Plastic Surgery, Department of Surgery, Beth Israel Deaconess Medical Center, Harvard Medical School, Boston, MA 02215, USA; jfoppian@bidmc.harvard.edu (J.A.F.); itarits1@bidmc.harvard.edu (I.T.); ahernan8@bidmc.harvard.edu (A.H.A.); dlee23@bidmc.harvard.edu (D.L.); mescoba2@bidmc.harvard.edu (M.J.E.-D.); kaschust@bidmc.harvard.edu (K.A.S.); sjlin@bidmc.harvard.edu (S.J.L.); 2 Institute of Pathological Physiology, First Faculty of Medicine, Charles University, 12108 Prague, Czech Republic; 3 Eberhard Karl University of Tübingen, 72076 Tübingen, Germany; josephine.berger@student.uni-tuebingen.de; 4 First Department of Internal Medicine, Department of Hematology, First Faculty of Medicine Charles University, General University Hospital, 12808 Prague, Czech Republic; 5 MD Anderson Cancer Center, The University of Texas, Houston, TX 77030, USA; mwclemens@mdanderson.org

**Keywords:** BIA-ALCL, biofilms, bacterial etiology, breast implants, molecular pathway

## Abstract

Breast implant-associated anaplastic large cell lymphoma (BIA-ALCL) is a distinct subtype of T-cell non-Hodgkin lymphoma that arises in the context of prolonged exposure to textured breast implants. The intent of this manuscript is to explore whether the bacterial presence in biofilms on these implants is a mere incidental finding or plays a pivotal role in the pathogenesis of BIA-ALCL. Our goal is to delineate the extent of bacterial involvement, offering insights into potential underlying mechanisms, and establishing future research priorities aimed at resolving the remaining uncertainties surrounding this complex association. A comprehensive systematic review of several databases was performed. The search strategy was designed and conducted by an experienced librarian using controlled vocabulary with keywords. The electronic search identified 442 publications. After evaluation, six studies from 2015 to 2021 were included, encompassing 201 female patients aged 23 to 75. The diagnosis span post-implantation ranged from 53 to 135.6 months. Studies consistently found bacteria near breast implants in both BIA-ALCL cases and controls, with varied microbial findings. Both BIA-ALCL cases and controls exhibited the presence of specific bacteria, including *Pseudomonas aeruginosa*, *Klebsiella oxytoca*, *Staphylococcus aureus*, and *Ralstonia* spp., without any statistically significant differences between groups. The use of antiseptic and antimicrobial agents during implant insertion did not demonstrate any impact on reducing or altering the risk of developing BIA-ALCL. Our systematic review reveals that the current evidence is inadequate to link bacterial etiology as a central factor in the development of BIA-ALCL. The limitations in the existing data prevent a complete dismissal of the role of biofilms in its pathogenesis. The observed gap in knowledge underscores the need for more focused and comprehensive research, which should be structured in a multi-faceted approach. Initially, this involves the utilization of sophisticated genomic and proteomic methods. Following this, it is crucial to delve into the study of immunological reactions specifically induced by biofilms. Finally, this research should incorporate extended observational studies, meticulously tracking the evolution of biofilm development and its correlation with the emergence of BIA-ALCL. In light of the inconclusive nature of current findings, further investigation is not only justified but urgently needed to clarify these unresolved issues.

## 1. Introduction

Breast implant-associated anaplastic large cell lymphoma (BIA-ALCL) is a type of T-cell non-Hodgkin lymphoma (NHL) that arises around breast implants, specifically in textured prostheses. This uncommon and emerging entity of NHL was first described by Keech and Creech in 1997 but it was not until 2016 that the World Health Organization (WHO) acknowledged it as a type of NHL [1,2].

According to the FDA as of April 2022, there is a prevalence of 1130 worldwide cases of BIA-ALCL with 59 deaths [3]. Of the total documented cases with a complete clinical history, by definition, 100% of cases involved prior exposure to textured surface implants or tissue expanders, with a median age of diagnosis of 53 years and a median time to diagnosis of 8 years [3]. Clinically, most recent statistics report that BIA-ALCL presents in 49% of cases with seroma formation, followed by non-specific symptoms and swelling in 32% and 23% and only presenting as a lump in 11% of the cases [3]. The risk of BIA-ALCL varies according to the manufactured risks but the American Society of Plastic Surgeons has estimated a 1:2207 to 1:86,029 lifetime risk of BIA-ALCL for women with textured implants based upon current confirmed cases and textured implant sales data over the past two decades [4,5].

The pathogenesis involved in the development of BIA-ALCL is still not completely understood. However, several possible mechanisms have been proposed. Among the most commonly cited hypotheses are genetic predisposition (i.e., DNMT3A, JAK-STAT3 pathway, and mutations in TP53), bacterial biofilm (BF), chronic inflammation, and digestion of particulate debris shed from textured breast implants [6].

The investigation into the role of BF in the development of BIA-ALCL continues to captivate a diverse array of stakeholders, including surgeons, regulatory authorities, manufacturers, and those involved in litigation efforts [7]. Existing research suggests that the textured surface of breast implants may facilitate biofilm formation, fostering bacterial growth and eliciting an enhanced T-cell response. This bacterial proliferation can lead to chronic inflammation, which, in individuals with certain genetic predispositions, may trigger a malignant transformation resulting in BIA-ALCL [7]. In light of these findings, the primary aim of this manuscript is to conduct a thorough examination of the existing evidence regarding the relationship between biofilm formation on breast implants and the onset of BIA-ALCL. By investigating these aspects, we seek to clarify the extent to which biofilms contribute to the pathogenesis of BIA-ALCL and to identify critical areas for future research in understanding and addressing this complex medical challenge.

## 2. Method

This study protocol was prospectively registered with PROSPERO (York, UK) (Study#: CRD42023424348) [8,9]. Completion of this study was performed in accordance with the Preferred Reporting Items for Systematic Reviews and Meta-Analysis (PRISMA) statement guidelines [8,9].

### 2.1. Eligibility Criteria

Criteria for included studies were defined as any studies investigating the relationship between bacteria or bacterial biofilm and the formation of BIA-ALCL in experimental or human settings. The full eligibility criteria are accessible at PROSPERO and are as follows:

Inclusion criteria:

- Female adult population 18 years and above
- Female with BIA-ALCL or suspicion of BIA-ALCL
- Animal, cadaveric, or experimental studies on BIA-ALCL
- Studies on BIA-ALCL investigating BF or bacteria
- Studies in English and studies translated to English

Exclusion criteria:

- Editorials, commentary reports, abstracts, and letters to the editors
- Studies conducted in patients with history of non-Hodgkin lymphoma or Hodgkin lymphoma

### 2.2. Search Strategy

A comprehensive research review using subject headings, controlled vocabulary, and keywords was conducted on 10 June 2023, on MEDLINE (in Ovid), Web of Science, and the Cochrane Central Register for studies published until June 2023. Our full-text search strategy is accessible at PROSPERO [8,9].

### 2.3. Study Selection

The search results were uploaded into the online systematic review program Covidence to conduct study selection [8,9]. Five independent reviewers performed a two-stage screening process for study selection. First, titles and abstracts were screened. A third reviewer then moderated if discordances were present and resolved any conflicts. Next, a full-text analysis was performed by the same five reviewers. If conflicts arose between reviewers, a third reviewer moderated a discussion to come to a joint decision.

### 2.4. Data Extraction/Synthesis

Data extraction was guided by a predetermined checklist: author, year of publication, type of study, total population, total samples, age of population, time till diagnosis, total number of BIA-ALCL cases, type of implants, method for biofilm analysis, and results.

### 2.5. Outcomes

These results of this systematic review focused on gathering the evidence in the literature for the biofilm induced theory of BIA-ALCL. This includes the type, colony forming unit, and other analysis performed in this area.

### 2.6. Quality Assessment

To assess the risk of bias, the National Institute of Health (NIH) quality assessment tool was utilized [8,9,10]. Each article was categorized as follows: “low risk”, “moderate risk”, or “high risk” of bias. All six studies were found to have a low risk of bias.

### 2.7. Statistical Analysis

Due to the heterogenicity of the topics covered in the studies constituting this systematic review, it was not possible to perform any analysis beyond a qualitative synthesis [11,12,13,14].

## 3. Results

**Figure 1:**
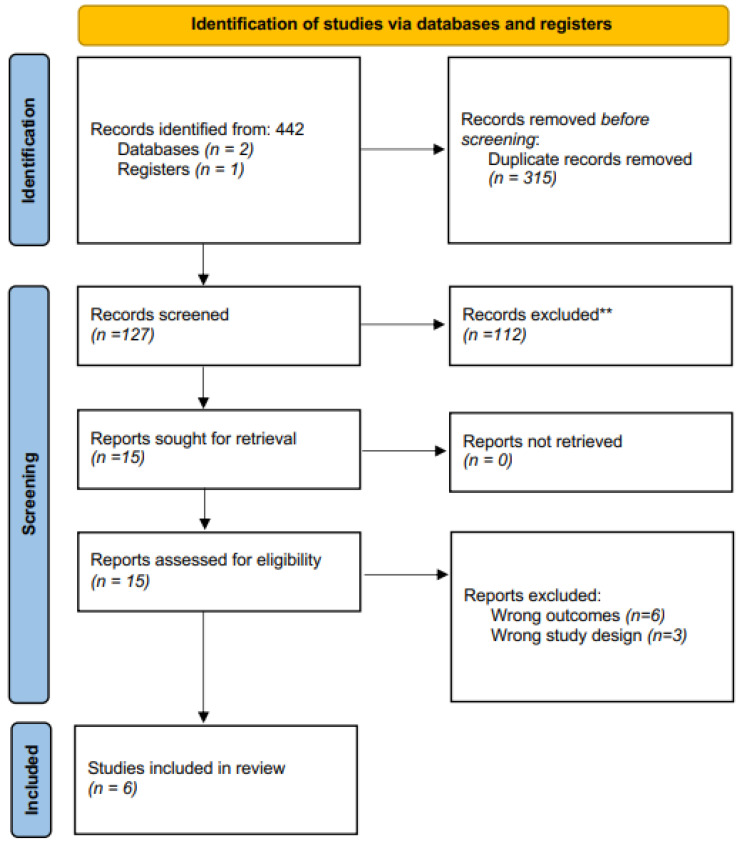
Prisma flow chart. ** Records excluded based on the abstract.

The electronic search initially identified 442 related publications, which ultimately yielded 14 studies for full-text review (Figure 1). After thorough assessment and subsequent exclusion, six studies from 2015 to 2021 were included for qualitative analysis (Table 1). These consisted of 3 clinical studies, 2 experimental studies, and 1 mixed study. Figure 1 Prisma flow chart [7,15,16,17,18,19].

### 3.1. Results—Clinical

A total of 201 female patients were included. The age range was 23 to 75 years old. Among this cohort, 60 were confirmed to have BIA-ALCL. Among these patients, the time to diagnosis ranged from 53 to 135.6 months from breast implant implantation [7,15,16,17,18,19].

#### 3.1.1. Summary of Method—Clinical

In the pursuit to elucidate the potential relationship between bacteria and the development of BIA-ALCL, several studies have charted a detailed methodological landscape. Central to the research efforts of Di Napoli et al., Hu et al. (2015 and 2016), and Walker et al. is the extraction and evaluation of bacterial DNA from breast implant samples, leveraging the 16S rRNA gene as a key indicator for microbiome sequencing [7,15,16,17,18,19].

Each investigation utilized a combination of proteinase K digestion and lysozyme treatments, paired with phenol/chloroform extraction for DNA isolation. Additionally, all methodologies incorporated real-time quantitative polymerase chain reaction (qPCR) techniques, albeit with subtle variances in reaction mixes and cycling conditions. This common methodology underscores a cohesive scientific strategy to probe the bacterial milieu potentially associated with BIA-ALCL [7,15,16,17,18,19].

Despite a shared objective, the investigative methodologies in the study of BF and their role in BIA-ALCL reveal nuanced differences. Di Napoli et al. primarily utilized culture-based techniques, employing a range of agar media to facilitate bacterial growth detection. In a marked contrast, Hu et al. (2015) adopted scanning electron microscopy and confocal imaging for direct visualization of biofilms. Their subsequent study in 2016 further expanded the scope by integrating pyrosequencing and fluorescence in situ hybridization (FISH) for a more comprehensive analysis of bacterial species. Walker et al., meanwhile, uniquely focused on the use of sonication, coupled with Sanger sequencing, to pinpoint specific bacterial colonies [7,15,16,17,18,19].

These studies, while converging towards a common goal, showcase a diversity of techniques—both overlapping and distinct. This variation underscores the complex and multifaceted nature of researching bacterial associations with BIA-ALCL in a clinical setting. However, it is important to acknowledge that such methodological disparities pose challenges in directly comparing results, thereby adding a layer of complexity to the interpretation and understanding of the findings (Table 2) [7,15,16,17,18,19].

#### 3.1.2. Summary of Results—Clinical

All five studies aimed to elucidate the relationship between bacteria and the onset of BIA-ALCL. They consistently found bacteria near the breast implants in BIA-ALCL cases but also in all controls. For instance, Di Napoli et al. identified pathogens in one mixed-type seroma and in four acute-type effusions. Hu et al. (2015) found that all periprosthetic capsules from their cohort were positive for biofilm bacteria, averaging 2.52 × 10^7^ bacteria/mg of a capsule. In Hu et al. (2016), the average bacterial count stood at 4.7 × 10^6^ bacteria/mg of tissue for BIA-ALCL and 4.9 × 10^6^ for nontumor capsule specimens. However, results from this study were limited by variability in specimen handling and preservation methods such as fresh versus formalin-fixed samples, absence of data on patients’ concurrent antibiotic treatments, and the detection of *Ralstonia* spp. in both BIA-ALCL and non-BIA-ALCL capsules. The predominant microbiome in both tumor and non-tumor capsules was characterized by *Staphylococcus* spp. Walker et al. revealed the presence of *Staphylococcus* spp. in both BIA-ALCL and controls [15,16,17,18,19].

A shared finding across the board was the association between immune response and bacteria. Both Di Napoli et al. and Hu et al. (2015) discussed the presence of CD30+ atypical cells. Hu et al. (2015) also recorded significantly more T cells than B cells, with correlations between bacterial load and lymphocyte count (e.g., CD4, r = 0.83) [7,15,16,17,18,19].

The studies varied in their clinical focus and depths of microbial investigation. Di Napoli et al. presented clinical details, like the BIA-ALCL localization to the fibrous capsule and treatments such as surgery or adjuvant chemotherapy. They found that culture was positive for *Pseudomonas aeruginosa* and *Klebsiella oxytoca* in one mixed-type seroma, and *Staphylococcus aureus* and *Serratia marcescens* in acute-type effusions [7,15,16,17,18,19]. Hu et al. (2015) drew correlations between implant texture and bacterial colonization; for example, that polyurethane implants harbored significantly more bacteria than other textures (*p* < 0.005) [7,15,16,17,18,19]. Hu et al. (2016) introduced deeper microbial insights, with BIA-ALCL and contralateral breast specimens predominantly showing *Ralstonia* spp. (*p* < 0.05), whereas nontumor capsules had a higher presence of *Staphylococcus* spp. (*p* < 0.001). Furthermore, they were able to visualize bacteria in 10 out of 11 samples (one BIA-ALCL sample was absent bacteria) using fluorescent in situ hybridization [7,15,16,17,18,19]. It is noteworthy that *Ralstonia pickettii*, a spirochete often a contaminant in laboratory water sources, was identified in this study. The implications of this finding, particularly in relation to potential laboratory contamination or environmental influences, warrant further exploration and consideration in the context of this study’s conclusions.

Walker et al. took a broader approach by comparing BIA-ALCL to multiple control types, including contralateral and cosmetic controls. They identified *Propionibacterium* as a significant driver of variability and noted that *Ralstonia* was found at low abundance in eight samples and that it was primarily identified in non-BIA-ALCL controls [7,15,16,17,18,19].

In a prospective study reported by Decoster et al. involving BIA-ALCL, details of intraoperative techniques used during the original breast implant placement were examined in 24 patients [7,15,16,17,18,19]. Among these patients, 12 underwent betadine irrigation (at varying strengths: 6 at 50%, 4 at 25%, and 2 with “tea-colored” solution), and 7 received antibiotic irrigation (5 with a combination of bacitracin/cefazolin/gentamicin and 2 with polymyxin/bacitracin). Notably, these patients developed BIA-ALCL despite these antimicrobial precautions. BIA-ALCL still occurred despite stringent antimicrobial measures. Globally, Decoster reported 18 clusters of BIA-ALCL, each involving two or more cases attributed to the same surgeon [7,15,16,17,18,19].

This study revealed that in only five clusters, the surgeon involved was the one who originally implanted the device. In the remaining cases, surgeons were handling secondary referrals. Four of the surgeons had previously contributed to peer-reviewed literature on BIA-ALCL. These surgeons represent established implant practices known for rigorous follow-up protocols, a low threshold for CD30 screening of seromas, and where feasible, lifelong annual surveillance of patients [7,15,16,17,18,19].

To sum up, whereas all studies consistently identified bacteria in proximity to breast implants, they varied in their specific microbial findings, clinical details, and correlations. The findings broadly reveal that bacteria are present in both BIA-ALCL and non-BIA-ALCL cases, which raises questions regarding a direct causal link between biofilm and this malignancy. Nevertheless, the data also emphasize the significant role bacteria play in inflammatory processes, contributing to the complex landscape of potential causative factors for this condition. No operative technique involving bacterial control has been shown to reduce the risk of developing this disease (Table 3) [7,15,16,17,18,19].

### 3.2. Results—Experimental

In the realm of experimental research, Hu et al. (2015) meticulously employed porcine models to delve deeper into the intricate relationship between the textures of implants—specifically distinguishing between smooth and textured varieties—and the associated bacterial colonization in the context of BIA-ALCL. Mempin et al. demonstrated functional differences between BIA-ALCL patient-derived primary cells and three BIA-ALCL cell lines, compared to T cells isolated from patients’ PBMC and cutaneous ALCL cell lines. Based on their observations, Mempin et al. proposed a unique BIA-ALCL pathogenesis, in which bacterial lipopolysaccharide promotes BIA-ALCL cell proliferation via a toll-like receptor 4 [7,15,16,17,18,19].

#### 3.2.1. Summary of Method—Experimental

Both Hu et al. (2015) and Mempin et al. investigate bacterial interactions with cell populations within the BIA-ALCL framework. A striking similarity is their utilization of advanced laboratory methodologies. Specifically, Hu et al. employed real-time qPCR, targeting the eubacterial 16 rRNA gene, to quantify bacterial numbers and discern specific T and B cell populations using genes like CD3, CD4, CD8a, and CD79a. Their approach aimed to comprehend bacterial colonization on implants and used confocal microscopy with monoclonal antibodies to visualize lymphocytic infiltrates on breast implant surfaces [7,15,16,17,18,19].

Conversely, Mempin et al. centered their research on cellular reactions to bacterial-secreted elements, particularly stimulants like PHA, LPS, SEA, and TSST-1. Their emphasis was on delineating apoptotic events in cells. To this end, they employed advanced staining and gating techniques, notably Zombie UV and Annexin V-FITC, to categorize cells based on their apoptotic states [7,15,16,17,18,19].

In essence, whereas both papers pivot on the bacterial-cell interface in BIA-ALCL, their research angles differ. Hu et al. delve deeper into bacterial colonization, its interaction with tissues, and their visualization, whereas Mempin et al. prioritize the cellular apoptotic responses upon exposure to bacterial-derived factors (Table 4) [7,15,16,17,18,19].

#### 3.2.2. Summary of Results

Both the study by Hu et al. (2015) and the research from Mempin et al. predominantly revolved around the intricate links between bacterial presence and BIA-ALCL, yet they approached the issue with varying focuses and depths [7,15,16,17,18,19].

Hu et al. (2015) concentrated on discerning the impact of implant texture on bacterial colonization and immune response. Their analysis comprised 10 capsular specimens surrounding both smooth and textured implants. Although there was no significant difference in bacterial load per milligram between capsules surrounding different implant types, textured implants were found to harbor significantly more bacteria than smooth ones (*p* < 0.001). Moreover, textured implants exhibited a staggering 63-fold increase in lymphocytes on their surface, predominantly T cells, in comparison to their smooth counterparts. This observation was validated by scanning electron microscopy which showcased numerous activated lymphocytes on the surface of textured implants, revealing a clear association between bacterial colonization and heightened immune response [7,15,16,17,18,19].

In contrast, Mempin et al. delved deeper into the cellular response of BIA-ALCL tumor cells and related cell lines upon exposure to bacterial stimulants. Their findings underscored that these cells exhibited a pronounced response to LPS stimulation over other bacterial agents like SEA and TSST-1. Remarkably, whereas LPS stimulation did amplify the BIA-ALCL and TLBR live cell number (*p* < 0.05), it did not significantly influence cell viability or the onset of apoptosis. This underscores a nuanced interplay where bacterial agents like LPS can boost cell proliferation without impacting their survival rates or leading them toward apoptosis [7,15,16,17,18,19].

In essence, both papers focus on differing aspects—Hu et al. (2015) on implant textures and Mempin et al. on cellular responses to specific bacterial agents—yet collectively, their results showcase the impact of bacteria on the immune system, not directly on the pathogenesis of BIA-ALCL. Table 5, summary of results of the experimental studies [7,15,16,17,18,19].

## 4. Discussion

Our review indicates that bacteria are found in both control samples and BIA-ALCL cases, offering no compelling evidence to link bacterial contamination during breast implantation directly to BIA-ALCL. Although there appears to be a connection between biofilm formation, years after implantation and inflammatory responses, a direct relationship with BIA-ALCL remains unsubstantiated. No operative technique involving bacterial mitigation at the time of implantation has been shown to reduce the risk of developing this disease.

The bacterial species and presence of bacterial toxins produced in the microenvironment surrounding breast implant has been hypothesized to be an important factor in BIA-ALCL development. Interestingly, Walker et al., as opposed to Hu et al., did not find a gram-negative shift in BIA-ALCL samples, with especially greater amounts of *Ralstonia* spp. found in control as opposed to BIA-ALCL samples [16,17,19]. This diverges from certain hypotheses previously referred to in the literature, which have posited a higher abundance of Gram-negative bacteria in BIA-ALCL cases [16,17,20,21]. Mempin et al. did indeed find an association between gram-negative bacteria and a dysregulation of immunomodulation leading to the proliferation of malignant lymphocytes [18]. In their 16 patients with BIA-ALCL, lipopolysaccharide (LPS) stimulation significantly increased BIA-ALCL tumor cell proliferation (*n* = 11), and this response was unique to LPS. When compared with exposure to Staphylococcal superantigens SEA and TSST-1 or to PHA, patient-derived BIA-ALCL had significantly more response to LPS (*p* < 0.001, *p* < 0.01). This response was also unique to the tumor cells themselves, and the same proliferative reaction to LPS was not seen in the peripheral blood mononuclear cells from BIA-ALCL patients. These results from Mempin et al. suggest that Gram-negative bacterial strains, and specifically the LPS coating their outer surface, may stimulate a local tumor response but not a general systemic response which was notably abrogated in the presence of a Toll-like receptor 4 (TLR4) inhibitor peptide. The findings underscored a novel pathway through which LPS could propel BIA-ALCL cell proliferation via TLR4 receptors [16,17,18]. Although one might infer from Mempin et al. that Gram-negative bacteria have a tumorigenic effect, and even a stimulatory effect in the presence of BIA-ALCL tumor cells, the absence of a Gram-negative predominance in clinical BIA-ALCL samples calls into question the direct involvement of this tumorigenic pathway in the genesis of BIA-ALCL [22]. Additional studies demonstrating a Gram-negative predominance in the native biofilm milieu of BIA-ALCL samples would be required to corroborate this as a relevant clinical pathogenic pathway.

To further analyze the effect of bacterial exposure in the breast implant environment on BIA-ALCL, the Walker et al. study helped show that the relative abundance of Gram-negative bacteria had no apparent role on BIA-ALCL [19]. They also showed that diversity in the microbiota of the skin, breast, implant, and capsule had no identifiable correlation with BIA-ALCL samples versus controls. Unlike Hu et al., they failed to see significantly elevated *Ralstonia* spp. in the breast implant microenvironment after using 16S rRNA sequencing between control and BIA-ALCL. Another layer of complexity pooling on the relatively low evidence for and against the part that biofilm plays in BIA-ALCL is that *Ralstonia* spp. are well-known contaminants that have been found in molecular grade water and PCR reagents [23,24]. This suggests that determining the predominance or lack thereof of gram-negative bacteria based on the species *Ralstonia* spp. as a representative may be flawed. More research is needed into the question of whether there are specific strains associated with the development of BIA-ALCL, which should focus on utilizing another Gram-negative bacterial species instead of *Ralstonia* spp.

In alignment with the guidelines from the American Society of Plastic Surgeons (ASPS) and numerous other organizations, the meticulous irrigation utilizing solutions such as Triple antibiotic solution, Povidone-iodine, Betadine in varying concentrations, or hypochlorous acid, has been emphasized to avert infections in the context of breast augmentation or alloplastic breast reconstruction. Although this does not preclude the theory that biofilm occurs due to contamination during implantation, it makes it rather unlikely [25,26,27,28,29,30,31]. It is more probable that the propensity toward biofilm formation is orchestrated via bacterial seeding, through bacteremia or other mechanisms, of the implant over time within the patients, a scenario mirroring those in other pathologies linked with closed cavities [32,33,34,35]. Within the domain of plastic surgery, if the hypothesis of a strong bacterial linkage to BIA-ALCL was validated, it could markedly shape breast implant safety protocols. The existing practice of submitting seroma fluid for culture in implant scenarios is a judicious measure that is in line with the standard of care and recommendation for suspicion of BIA-ALCL [36,37]. If a bacterial-derived BIA-ALCL pathway was identified, we could amplify this practice of seroma evaluation and integrate capsule biopsy and culture during implant or expander exchange. The identification of particular bacterial species in these instances could help stratify patients as having an escalated risk for BIA-ALCL. The magnitude of the association between bacterial existence and BIA-ALCL could, in turn, guide the cadence and thoroughness of monitoring regimes. Moreover, the insights procured from such endeavors could potentially delineate the prerequisites for prophylactic capsulectomy, thereby forging a more individualized and evidence-informed approach to augmenting patient safety and diminishing BIA-ALCL risks.

Our systematic review on the association between BIA-ALCL and bacterial biofilm as a potential contributor is not without its limitations. A primary challenge encountered during this review was the limited number of studies that undertook bacterial sampling or testing within the available literature addressing BIA-ALCL. Given this scarcity, drawing a comprehensive and definitive correlation between BIA-ALCL and bacterial biofilms becomes inherently challenging. However, by aggregating the existing literature and evidence on this topic, our review has provided the most exhaustive insight on the subject to date. Additionally, the very nature of BIA-ALCL, and the extended latency period before the malignancy manifests, further underscores the need for better modeling in future research. As with any systematic review, there exists inherent heterogeneity between studies which, in this case, was further accentuated by the specificity of bacterial testing in relation to BIA-ALCL. This variability prevented a more unified and meta-analytic approach to the results. Despite these limitations, our review has shed critical light on the relationship between bacterial biofilms and BIA-ALCL. Additionally, the scope of this systematic review was confined to three medical databases. Although these databases are extensive, this limitation may have led to the exclusion of certain relevant data.

## 5. Conclusions

Our systematic review reveals an absence of compelling evidence to substantiate a direct link between bacterial biofilms and the pathogenesis of BIA-ALCL within the milieu of breast implants. Although current data intimate a potential contributory role for biofilms, these assertions remain uncorroborated. This observed data scarcity necessitates further targeted, rigorous scientific inquiry to either validate or refute the role of biofilms in the complex etiological framework of BIA-ALCL. Such forthcoming research could serve as a critical juncture in not only clarifying this enigmatic relationship but also in advancing our collective understanding of the disease, with implications for enhanced screening protocols and pre-surgical guidelines. Given the inconclusive nature of extant findings, further empirical scrutiny is both warranted and exigent. Given the current data limitations and inconclusive findings, continued exploration is both imperative and timely.

## Figures and Tables

**Table 1:** Study characteristics.

Title	First Author Last Name/Year	Type of Study	Total Population	Total Sample	Age of Population (Mean)	Time till Diagnosis (Years)	Total Case of BIA-ALCL	Type of Implants	NIH Quality Assessment
Cytological diagnostic features of late breast implant seromas: From reactive to anaplastic large cell lymphoma	Di Napoli, 2017 [15]	Clinical	50	67	23–75	5.83 ± 1.42	5	4 Biocell texture silicon implants, 1 Polyurethane- coated silicone	Good
Chronic biofilm infection in breast implants is associated with an increased T-cell lymphocytic infiltrate: implications for breast implant-associated lymphoma.	Hu, 2015 [16]	In Vivo (pig)	3	24	N/A ^*^	0.73	0	12 textured vs. 12 smooth	Good
		Clinical	34	57	N/A	N/A	0	Smooth implant	
Bacterial Biofilm Infection Detected in Breast Implant-Associated Anaplastic Large-Cell Lymphoma.	Hu, 2016 [17]	Clinical	84	88	52.7 (BIA-ALCL only), 43.8 (capsule patients)	8.8 (BIA-ALCL only), 7.4 (capsule patients)	22	13 textured, 9 unknown	Good
Gram-Negative Bacterial Lipopolysaccharide Promotes Tumor Cell Proliferation in Breast Implant-Associated Anaplastic Large-Cell Lymphoma.	Mempin, 2021 [18]	In vitro	Tumor cell line: 16 patients PBMC: 12 patients with BIACL, 3 controls	N/A	43.8 years	7	16	8 Silimed polyurethane (textured), 6 Allergan Biocell (textured), 1 Mentor Siltex (textured), 2 Nagor, 1 McGhan, 2 polyimplant prostheses	Good
Insights into the Microbiome of Breast Implants and Periprosthetic Tissue in Breast Implant-Associated Anaplastic Large Cell Lymphoma.	Walker, 2019 [19]	Clinical	9	58	51.7 ± 11.4 years	9.3 ± 2.0	7	Textured implants	Good
Breast Implant-Associated Anaplastic Large Cell Lymphoma: Defining Future Research Priorities	DeCoster, 2021 [7]	Clinical	24	18	N/A	1–28	18	Textured silicon implants	Good

* Information not available (N/A).

**Table 2:** Clinical studies methodology.

Title	Method Biofilm/BIA-ALCL Identification
Cytological diagnostic features of late breast implant seromas: From reactive to anaplastic large cell lymphoma [15]	From 61 reactive late seromas, 21 samples underwent aerobic, anaerobic, and fungal cultures, incubated in a brain–heart infusion broth and fluid thioglycolate medium, then spread on various agar plates. Post 24 h incubation at 37 °C, samples with no bacterial growth after 48 h were deemed negative. Cell-block and FFPE specimens underwent immunohistochemistry and molecular T-cell receptor analysis, with PCR products analyzed via a genetic analyzer. Capsulectomy specimens from five BI-ALCL patients were collected post-diagnosis.
Chronic biofilm infection in breast implants is associated with an increased T-cell lymphocytic infiltrate: implications for breast implant-associated lymphoma [16]	Capsule or implant samples (50 or 100 mg) were digested using proteinase K and lysozyme, followed by genomic DNA extraction via phenol/chloroform and ethanol precipitation. Bacterial counts were determined by qPCR of the 16S rRNA gene. T- and B-cell quantities in capsules or on implants were ascertained by qPCR of specific genes. Bacteria and lymphocyte counts were calculated per milligram of capsule. Real-time qPCR involved a mix with cycling conditions of 95 °C for 10 min, followed by 40 cycles. Biofilm presence was confirmed by scanning electron microscopy, and lymphocytic infiltration was analyzed using monoclonal antibodies and confocal laser scanning microscopy.
Bacterial Biofilm Infection Detected in Breast Implant-Associated Anaplastic Large-Cell Lymphoma [17]	Samples (50–100 mg) from 26 breast implant-associated ALCL and 62 non-tumor capsules were digested using proteinase K and lysozyme, followed by genomic DNA extraction. Bacterial DNA was quantified using qPCR of the 16S rRNA gene, with the 18S rRNA gene as a reference. Bacterial count per milligram of tissue was based on the 18S rRNA gene’s average copies. Real-time qPCR involved a 25 μL mix with specific cycling conditions. Bacterial species were identified in 19 ALCL and 12 non-tumor samples, plus 3 contralateral breast samples, using FLX amplicon pyrosequencing and analyzed by QIIME software (Ver 1.3.0) (Boulder, CO, USA). The bacterial composition and abundance were compared using one-way ANOVA. Non-tumor capsules were fixed in glutaraldehyde, and ALCL samples were prepared from formalin-fixed, paraffin-embedded blocks, dehydrated, and examined under a scanning electron microscope. Bacterial aggregates were located using fluorescence in situ hybridization with specific probes.
Insights into the Microbiome of Breast Implants and Periprosthetic Tissue in Breast Implant-Associated Anaplastic Large Cell Lymphoma [18]	In each case of breast implant-associated anaplastic large-cell lymphoma (BIA-ALCL) and non-BIA-ALCL, an en bloc capsulectomy was performed. Specimens, including implant, capsule, skin, and parenchyma, underwent 16S rRNA microbiome sequencing, culturing, and imaging. Tissue and capsule samples were incubated for over 24 h at 37 °C. Visible microbial growth was isolated using a loop on BHI agar, with colonies identified through 16S Sanger sequencing. Homogeneous and heterogeneous colonies were processed for aerobic and anaerobic growth, with selected colonies sequenced. Biofilm samples were frozen for DNA extraction using the DNeasy PowerBiofilm kit.
Breast Implant-Associated Anaplastic Large Cell Lymphoma: Defining Future Research Priorities [7]	The diagnosis of breast implant-associated anaplastic large-cell lymphoma (BIA-ALCL) involves three main steps. First, CD30+ cells are identified using immunohistochemistry, but this alone is not conclusive for BIA-ALCL as such cells can also be present in benign seromas. Second, flow cytometry is used to assess T-cell clonality, crucial for confirming T-cell lymphoma. Finally, histological examination is performed to detect large anaplastic lymphoma cells, a key factor in diagnosing BIA-ALCL. In cases without obvious lesions, a thorough approach involving multiple biopsies from different capsule areas is recommended to enhance diagnostic accuracy.

**Table 3:** Clinical studies summary of results.

Title	Results
Cytological diagnostic features of late breast implant seromas: From reactive to anaplastic large cell lymphoma [15]	The lymphoma remained within the fibrous capsule around the implant, not spreading to nearby tissues. Only one case showed lymph node involvement at diagnosis. Four patients underwent surgical removal of the implant and capsule, whereas the fifth, with lymph node involvement, also received adjuvant chemotherapy, leading to complete remission. About 70% of cells in these samples were CD30-positive atypical neoplastic cells. One patient had a relapse, evidenced by a seroma containing mixed cells, including 10% large atypical cells. All cases showed a T-cell defective phenotype, predominantly CD4+, except for one CD8+ and one double-negative. Four cases presented monoclonal TRG rearrangement, and one was polyclonal. Of 61 seromas tested, 21 were cultured, showing various types including acute and mixed; 1 mixed seroma grew *Pseudomonas aeruginosa* and *Klebsiella oxytoca*, and 4 acute seromas grew *Staphylococcus aureus* and Serratia marcescens. Chronic and hemorrhagic seromas showed no pathogen growth.
Chronic biofilm infection in breast implants is associated with an increased T-cell lymphocytic infiltrate: implications for breast implant-associated lymphoma [16]	In a four-year study, 57 periprosthetic capsules from 34 patients with Baker grade IV contracture were collected. All patients had textured implants. The capsules were analyzed using quantitative polymerase chain reaction to determine lymphocyte count, CD status, and bacterial count. All capsules contained biofilm bacteria, averaging 2.52 × 10^7^ bacteria/mg of tissue. Implant textures varied: 34 Biocell, 14 Siltex, 5 Poly Implant Prothèse, and 4 polyurethane. T cells (CD4 + CD8a) outnumbered B cells significantly, and a correlation was noted between lymphocyte and bacterial count per milligram of tissue. Polyurethane implants showed a notably higher bacterial presence compared to other textures.
Bacterial Biofilm Infection Detected in Breast Implant-Associated Anaplastic Large-Cell Lymphoma [17].	In a breast implant-associated ALCL study, five samples showed no bacteria via real-time quantitative polymerase chain reaction, with a significant inhibition of the human 18S rRNA gene’s reaction, suggesting PCR inhibitors’ presence. One negative sample, part of a pair from the same tumor, contrasted with its counterpart showing normal gene augmentation. The other 21 ALCL and 62 non-tumor samples displayed considerable bacterial 16S rRNA gene presence, averaging 4.7 × 10^6^ and 4.9 × 10^6^ bacteria/mg of tissue, respectively. Samples from the contralateral breasts had significantly fewer bacteria. Microbiome analysis of 19 ALCL samples revealed a predominance of *Ralstonia* spp., also noted in contralateral samples, whereas Staphylococcus spp. was common in non-tumor specimens. Fluorescent in situ hybridization in 11 ALCL samples detected *Ralstonia* spp. in 10, confirmed in half, and identified in all via pyrosequencing.
Insights into the Microbiome of Breast Implants and Periprosthetic Tissue in Breast Implant-Associated Anaplastic Large Cell Lymphoma [19]	*Staphylococcus* spp., frequently found in cultures, was identified in both BIA-ALCL and control samples from the contralateral breast. The diversity and relative abundance of Gram-negative bacteria were similar between BIA-ALCL and control samples. Heat maps showed significant variations in bacterial composition across different materials like skin, breast, implant, and capsule. When comparing BIA-ALCL to control samples by material, Propionibacterium was the main driver of variability, alongside *Staphylococcus*, Altererythrobacter, and Stenotrophomonas, although this varied across sample types. Rare bacterial taxa, including *Ralstonia* in 8 samples and Brevundimonas in 31 samples, were present but did not differentiate BIA-ALCL samples from controls. *Ralstonia* was more common in non-ALCL controls, whereas Brevundimonas showed higher abundance in some contralateral control and BIA-ALCL skin samples.
Breast Implant-Associated Anaplastic Large Cell Lymphoma: Defining Future Research Priorities [7]	In a study focused on breast implant-associated anaplastic large-cell lymphoma (BIA-ALCL), the intraoperative techniques used for breast implant placement in 24 patients were analyzed. Twelve of these patients underwent betadine irrigation at varying strengths, whereas seven were treated with different antibiotic irrigation combinations. Despite these measures, BIA-ALCL occurred in these patients. This study discovered 18 worldwide BIA-ALCL case clusters, each linked to a specific surgeon and involving multiple cases. Only in five clusters did the surgeon perform the original implantation, with the others involving secondary referral surgeons. Four of these surgeons had previously contributed to BIA-ALCL research and were recognized for their comprehensive implant practices, including diligent follow-up, proactive CD30 screening, and ongoing patient surveillance.

**Table 4:** Experimental studies methodology.

Title	Method Biofilm/BIA-ALCL Identification
Chronic biofilm infection in breast implants is associated with an increased T-cell lymphocytic infiltrate: implications for breast implant-associated lymphoma [16]	Pig tissue samples (50 or 100 mg) were digested with proteinase K and lysozyme, and genomic DNA was extracted using phenol/chloroform and ethanol precipitation. Bacterial count was determined by real-time quantitative polymerase chain reaction of the 16S rRNA gene. T- and B-cell counts in the tissue were quantified using qPCR of CD3, CD4, CD8a, and CD79a genes. Bacterial and lymphocyte numbers were calculated per milligram of tissue, based on the average 18S gene copies. The qPCR analysis involved specific reaction mix and cycling conditions. Biofilm presence was visually confirmed via scanning electron microscopy, and lymphocytic infiltration was imaged using confocal microscopy with specific monoclonal antibodies.
Gram-Negative Bacterial Lipopolysaccharide Promotes Tumor Cell Proliferation in Breast Implant-Associated Anaplastic Large-Cell Lymphoma [18]	BIA-ALCL and TLBR cells were exposed to Phytohemagglutinin (PHA), lipopolysaccharide (LPS), Staphylococcal enterotoxin A (SEA), and toxic shock syndrome toxin-1 (TSST-1) to study apoptosis induced by biofilm secretion factors. Apoptotic stages were identified using a bivariate dot plot of Zombie UV versus Annexin V-FITC expressions. The analysis distinguished viable cells (Annexin V and Zombie UV negative), early apoptotic (Annexin V positive, Zombie UV negative), dead, and late apoptotic/necrotic cells (both markers positive).

**Table 5:** Summary of the results of experimental studies.

Title	Results
Chronic biofilm infection in breast implants is associated with an increased T-cell lymphocytic infiltrate: implications for breast implant-associated lymphoma [16]	Ten capsular specimens from both smooth and textured implants were analyzed, with twenty samples undergoing quantitative polymerase chain reaction for bacterial 16S RNA gene detection. No significant difference was observed in bacterial count per milligram between smooth and textured implants (2.7 × 10^5^ vs. 3.5 × 10^5^ bacteria/mg). However, textured implants had significantly more attached bacteria (4.2 × 10^5^ bacteria/mg) than smooth implants (1.52 × 10^3^ bacteria/mg). Additionally, textured implants showed a higher lymphocyte count on their surface, with a 63-fold increase compared to smooth implants, mostly T cells. Scanning electron microscopy revealed numerous activated lymphocytes on textured implants, characterized by increased size and active replication, whereas smooth implants had minimal or no attached infiltrate.
Gram-Negative Bacterial Lipopolysaccharide Promotes Tumor Cell Proliferation in Breast Implant-Associated Anaplastic Large-Cell Lymphoma [18]	BIA-ALCL tumor cells and TLBR lines showed a stronger response to LPS than to Staphylococcal superantigens SEA, TSST-1, or PHA. Conversely, cutaneous ALCL cells and PBMCs from capsular contracture patients reacted more to PHA and Staphylococcal superantigens. Healthy control and BIA-ALCL patient-derived PBMCs also responded more to PHA. LPS stimulation increased the live cell count in BIA-ALCL and TLBR cells but did not affect cell viability or induce apoptosis. The proportions of live, dead, and apoptotic cells remained consistent across stimulated and non-stimulated cells, and between BIA-ALCL and TLBR cells.

## Data Availability

Data sharing not applicable. No new data were created or analyzed in this study. Data sharing is not applicable to this article. All information relevant to this systematic review is part of the manuscript, figures, tables, and/or digital supplemental content. Additional information can be found within the publicly available PROSPERO protocol for this study. If any further information is required, the reader may contact the corresponding author for clarifications.
